# The mechanism of knowledge-based behavior of pastoralists for rangeland management: exploitation, restoration and conservation

**DOI:** 10.1038/s41598-023-43590-0

**Published:** 2023-09-28

**Authors:** Seyedeh Khadijeh Mahdavi, Mohammadreza Shahraki, Mohsen Sharafatmandrad

**Affiliations:** 1 Department of Natural Resources, Nour Branch, Islamic Azad University, Nour, Iran; 2 Expert of the Department of Natural Resources and Watershed Management of Golestan Province, Gorgan, Iran; 3 Department of Ecological Engineering, Faculty of Natural Resources, University of Jiroft, 8th km of Jiroft - Bandar Abbas Road, P.O. Box: 7867161167, Jiroft, Iran

**Keywords:** Socioeconomic scenarios, Sustainability

## Abstract

Pastoralists have managed their lands for a thousand years, but they are ignored in the land management approaches. They have comprehensive information about their rangelands, coming from extensive observations and experiences in continuous herding. This research has focused on revealing the mechanism of knowledge-based behavior of pastoralists for rangeland management. The statistical population is made up of 50 pastoralists, all of whom were included in census. The research instrument was a researcher-made questionnaire that measured the knowledge-based behavior of pastoralists with 58 items in three indicators i.e. exploitation, conservation, and restoration. The validity and reliability of the research instrument were assessed using the opinions of local experts and Cronbach's alpha (α = 0.877). The knowledge-based behavior of pastoralists were from the highest to the lowest related to exploitation, conservation, and restoration with the average of 2.35, 2.07 and 1.58 respectively. Exploitation knowledge, restoration knowledge and conservation knowledge had the strongest and weakest significant relationship with the knowledge-based behavior of pastoralists. “The adequate growth of palatable plants is a sign of the start of grazing” and “the soil should not be wet for the start of grazing” had the highest importance for rangeland exploitation with a values of 0.816 and 0.784 respectively. For rangeland conservation, “holding meetings by elders regarding rangeland conservation is useful” and “reducing the number of pastoralists during droughts is one of the rangeland conservation ways” were the most importance items with the values of 0.852 and 0.848 respectively. For rangeland restoration, “implementation of grazing systems (rotation or rest rotation grazing systems) is one of the rangeland restoration ways” and “the appropriate distribution of watering points is one of the rangeland restoration factors” were the most importance items with the values of 0.840 and 0.812 respectively. There was a positive and significant relationship between the age, history of pastoralism and income with the knowledge-based behavior of pastoralists in rangeland management. Therefore, the presented approach based on indigenous knowledge can be considered as an effective component for rangeland management and can strengthen the positive effects of both management systems and create a transformation in the status of natural resources by a compatible combination of indigenous knowledge and modern knowledge. It is worth noting that by knowing these indicators, we can take an effective step in planning and policy making as well as proper management of rangelands.

## Introduction

Rangelands are one of the main land cover types on the globe, occupying about half of the Earth’s land area. Rangelands are of great economic and social importance, providing a livelihood, food security and poverty alleviation to millions of people^1^. They are home to 38% of the globe population^2^, supporting 50% of livestock of the world. Rangelands are mainly used for traditional animal husbandry in many developing countries^3^.Pastoralism is a special type of animal husbandry in arid and semi-arid regions, which is based on herding, mobility and opportunistic grazing of natural vegetation^4^. It is estimated that pastoralism is practiced by 50–500 million people worldwide^5,6^. There is no complete and accurate information about the origins of pastoralism, but it can be traced back to about 10,000 years ago in Zagros Mountains, Iran^7^.

Iran has long been a suitable platform for animal husbandry due to its special climatic and geographical conditions. With about 85 million hectares, rangelands are estimated to occupy 51.5% of the Iran's land area. Pastoralism is practiced by about 2 million people in Iran, supplying more than 25% of the country’ meat consumption. Pastoralists have gathered a vast information and knowledge about the environment, livestock and their grazing resources through extensive observation and repeated trials and errors, resulted from historic and continuous presence in rangelands^8^. The indigenous knowledge of pastoralists is essential for their natural resources management^9^. This knowledge has a significant impact on their management strategies and exploitation of natural ecosystem^10^. The indigenous knowledge of pastoralists and its documentation can play an important role for rangeland conservation and management^11,12^. Local pastoralists often have different perceptions about rangelands degradation^13^. The reinvestigation of rangeland health indicators based on the indigenous knowledge of pastoralists can be of great help in planning and policy making for sustainable management of rangelands^14^. The indigenous knowledge of pastoralists and scientific knowledge can be combined in sustainable management systems to reduce the degradation of rangelands. Despite the precious environmental knowledge of pastoralists, they are always ignored in policies and plans related to rangelands by governmental agencies^15^. The governmental top-down management based on formal knowledge and ignoring the pastoralists’ knowledge and views on rangeland management have deteriorated rangelands condition and destroyed pastoralists’ socio-economic self-sustaining systems, forcing many of them to leave their lands and resettle in urban centers. Over the past decades, science has played a key role in knowing the rangeland degradation indicators^16^, but it has always faced a series of limitations in providing appropriate strategies and solutions in planning and decision making. This is while formal knowledge and indigenous knowledge can be both used to more effectively improve the sustainability of rangelands and their management. The hypothetical role of pastoralists’ exploitation in land degradation is mainly based on beliefs induced by ecologists, therefore, the ecological knowledge of pastoralists, which is claimed to be non-scientific, has also been neglected^17^. The indigenous knowledge of pastoralists may help to gain better understanding of rangelands, support bottom-up pastoralists’ initiatives and discussions on sustainable land management and develop locally relevant global and national policies^10^.

**Figure 1:**
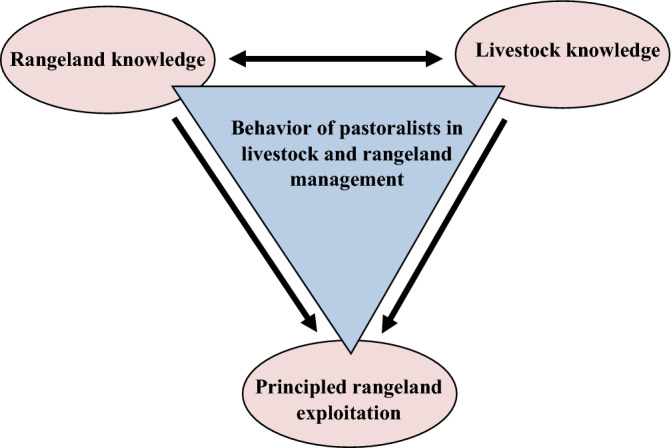
Relationships between pastoralists’ knowledge-based behavior for livestock and rangeland management.

On one hand, governmental rangeland management in Iran is technically based on the three main dimensions: conservation, restoration and principled exploitation. On the other hand, Iranian pastoralists from both nomadic and rural communities have rich indigenous knowledge for rangeland management. Considering the large area of rangelands and their role in sustainable supply of ecosystem services, it is important to plan rangeland management programs based on the indigenous knowledge of pastoralists. Connecting scientific knowledge with traditional ecological knowledge can fill existing gaps by bringing new perspectives^18,19^ and help the sustainable rangeland management. The pastoralists’ participation in decision-making for environmental monitoring and assessment is one of the basic principles of rangeland management^20^. Therefore the main objective of this study is to assess knowledge-based behavioral of pastoralists in three technical dimension of conservation, restoration and principled exploitation. Now the questions are, what are the behavioral phenomena of pastoralists in livestock and rangeland management based on their indigenous knowledge? And how effective are each of them in the principled conservation and exploitation of rangelands? Therefore, the present research aimed to reveal the knowledge-based behavioral indicators of pastoralists for rangeland management with the approach of indigenous knowledge (Fig. 1).

## Materials and methods

### Study area

**Figure 2:**
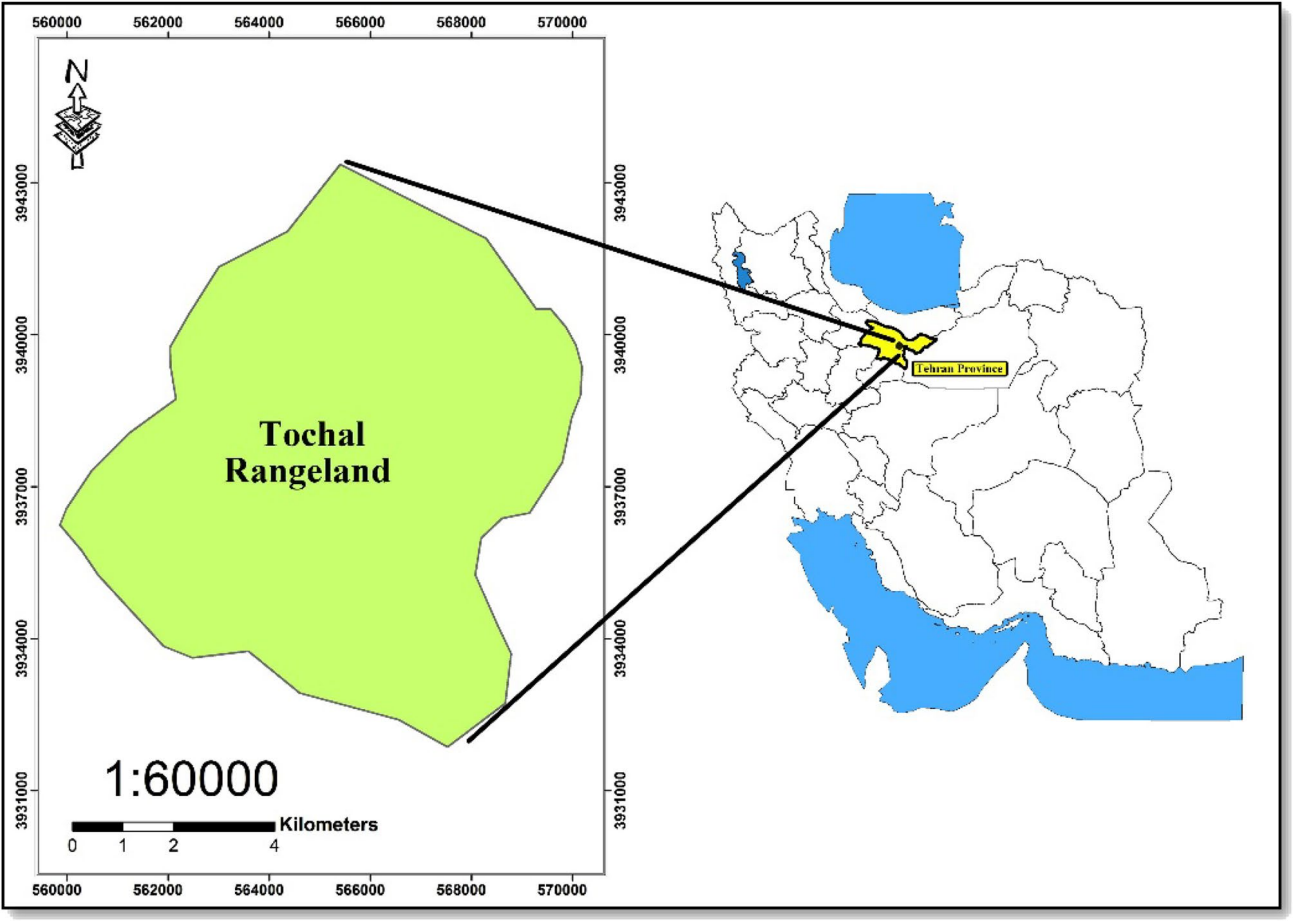
The location map of Tochal rangelands, Iran.

The studied area is Tochal rangelands located in Tehran province, Iran (3,931,785 N and 560,433 E to 3,942,868 N 570,709 E, Fig. 2). The region altitude ranges between 1300 and 2200 m a.s.l and has an area of 8133 hectares. The region rangelands are winter rangelands, which are used from the late October to April. Based on De Martonne climatic classification, the region climate is semi-arid. The hottest months of the year are July and August, when the absolute maximum temperature reaches 40 degrees Celsius, and the coldest months are January and February, with an absolute minimum of − 14 to − 16 °C.

### Data collection

In terms of practical purpose and information gathering, the present study is a descriptive research which is conducted in the field. The statistical population is made up of 50 pastoralists, all of whom were included in the census. The research instrument was a researcher-made questionnaire. The questionnaire items were set based on 10 individual interviews with expert pastoralists selected by snowball sampling. For the final confirmation of the items, two rounds of group interviews with 6 to 8 people were conducted in a centralized manner, which lasted for 480 min. Group interviews were conducted in the living places of pastoralists in the region. After summarizing the interviews, 23, 18 and 17 items were respectively finalized for exploitation, conservation, and restoration indicators. The respondents were then asked to express positive-to-negative strength of their agreement on each item on a five-point Likert scale including completely disagree (with a numerical value of 1), disagree (with a numerical value of 2), somewhat agree (with a numerical value of 3), agree (with a numerical value of 4) and completely agree (with a numerical value of 5) to measure the knowledge-based behavior of pastoralists with 58 items. The validity of the research instrument was assessed using the opinions of local experts and its reliability was also checked by calculating the Cronbach's alpha (α = 0.877). Data were analyzed by SPSS V.22. Frequency, frequency percentage, mean and standard deviation were used in order to describe personal qualities.

The relative importance index^21^ was used to prioritize items as follow:

$$Relative\; importance\; index = \frac{\sum w}{{AN}} = \frac{{1n_{1} + 2n_{2} + 3n_{3} + 4n_{4} + 5n_{5} }}{5N}$$

where w is the weight of each items provided by the respondents, varying from 1 to 5; n1 to n5 are the number of times a scale point was selected by the respondents (completely disagree, disagree, somewhat agree, agree and completely agree; A is the highest weight (= 5 in this study) and N is the total number of respondents (50 people). The relative importance index (w) ranges between 0 and 1.

In the inferential part, Friedman's test was used to compare the rank average and the importance of knowledge-based behavior of pastoralists. Spearman's correlation coefficient was used to investigate the relationship between personal qualities and the knowledge level of pastoralists.

### Ethics approval and consent to participate

All experimental protocols were approved by Review Board of Department of Natural Resources, Nour Branch, Islamic Azad University, Iran. All methods were carried out in accordance with relevant guidelines and regulations. Informed consent was obtained from all participants.

## Results

### Personal qualities

All the respondents were married men. Averagely, they were 57.46 years old. About 42% were aged between 56 and 65 years old with the highest frequency. Half of the respondents had more than 6 family members.Illiterate respondents made up more than half the population in the region. About 48% had reading and writing skills. The respondents averaged about 144.20 head of livestock per pastoral unit, ranged from 80 to 230 head of livestock. The average respondents' years of experience was about 41 years, ranged from 18 to 70 years (Table 1).

**Table 1:** Personal qualities of the respondents.

	Classes	Frequency	Frequency percentage	Mean	Standard deviation	Minimum	Maximum
Age	< 45	6	12	57.46	9.50	37	81
	46–55	15	30				
	56–65	21	42				
	66 <	8	16				
Family size	5	13	26	7.36	2.07	5	10
	6	12	24				
	6 <	25	50				
Literacy	Illiterate	26	52	–	–	–	–
	Elementary school	11	22				
	Guidance school	8	16				
	High school and higher	5	10				
Livestock number	< 100	15	30	144.20	42.84	80	230
	100–150	20	40				
	150 <	15	30				
Experience years in pastoralism	< 35	18	36	41.16	8.21	18	70
	35–45	12	24				
	45 <	20	40				

### The knowledge-based behavioral indicators of pastoralists

The prioritizing the items in knowledge-based behavioral indicators of pastoralists based on the relative importance index (Table 2) showed that “the adequate growth of palatable plants is a sign of the start of grazing” and “the soil should not be wet for the start of grazing” had the highest importance for exploitation indicator with the values of 0.816 and 0.784 respectively. For the conservation indicator, “holding meetings by elders regarding rangeland conservation is useful” and “reducing the number of pastoralists during droughts is one of the rangeland conservation ways” were the most importance items with the values of 0.852 and 0.848 respectively. For restoration indicator, “implementation of grazing systems (rotation or rest rotation grazing systems) is one of the rangeland restoration ways” and “the appropriate distribution of watering points is one of the rangeland restoration factors” were the most importance items with the values of 0.840 and 0.812 respectively.

**Table 2:** The ranking of the knowledge-based behavioral indicators of pastoralists.

Indicator	Item	Completely disagree	Disagree	Somewhat agree	Agree	Completely agree	Relative importance index	Rank
Exploitation	The adequate growth of palatable plants is a sign of the start of grazing	1	3	10	13	23	0.816	1
	The soil should not be wet for the start of grazing	2	5	8	15	20	0.784	2
	Rangeland depletion and dust emission due to livestock trampling are signs of the stop of grazing	4	3	13	19	11	0.720	3
	The soil should not be covered with snow for the start of grazing	3	3	9	19	16	0.768	4
	If keeping livestock in pen is not affordable, grazing begins sooner	2	4	10	21	13	0.756	5
	Preventing overgrazing is one of the ways to increase the use of rangelands	1	9	17	14	9	0.684	6
	The herd size should be at least 500 head of livestock to be profitable	10	6	9	18	7	0.624	7
	The area of grazing is determined by the elders	9	5	15	13	8	0.620	8
	The rangeland suitability depends on the rangeland accessibility (geographical situation)	10	6	15	9	10	0.612	9
	South-facing slopes are prepared earlier for grazing than north-facing slopes	5	10	15	19	1	0.604	10
	If rangeland condition is good, it can be grazed more	9	5	16	16	4	0.600	11
	In common grazing is better than individual grazing	7	8	20	10	5	0.592	12
	Night grazing is better than day grazing	11	6	12	16	5	0.588	13
	The soil type is effective on the rangeland utilization	7	9	22	5	7	0.584	14
	Climatic conditions are effective on the start and stop of grazing	10	5	25	6	4	0.556	15
	Stocking rate is determined based on the elders’ experience	15	4	14	10	7	0.560	16
	In the middle of the sunny and shady hillsides, there are intermediate slopes that are grazed after the sunny hillsides and before the shady hillsides	15	4	16	11	4	0.540	17
	If it snows well in a year and the weather warms up after the 5th of February, forage production will increase	13	4	24	5	4	0.532	18
	The destruction of intermediate rangeland is influential on the start and stop of grazing	12	4	26	5	3	0.528	19
	If forage plants reproduce, the grazing was principled	17	12	7	10	4	0.488	20
	Grazing get started by the orders of the elders	16	13	11	8	2	0.468	21
	The start of grazing depends on the health status of livestock	24	7	7	8	4	0.444	22
	Grazing begins based on the type and gender of livestock and the type of vegetation in different areas	26	8	2	9	5	0.436	23
Conservation	Holding meetings by elders regarding rangeland conservation is useful	0	2	11	10	27	0.852	1
	Reducing the number of pastoralists during droughts is one of the rangeland conservation ways	1	3	8	9	29	0.848	2
	Off-season overgrazing causes rangeland degradation	1	3	9	9	28	0.840	3
	Guaranteed purchase of livestock by government during droughts reduces grazing pressure	1	4	10	11	24	0.812	4
	Choosing the right herd composition and shifting animals between herds are useful for rangeland conservation	1	4	9	14	22	0.808	5
	Feed intake can regulated with salt	2	4	9	15	20	0.788	6
	Off-season grazing should be avoided	0	8	11	12	19	0.768	7
	As soon as wild rue (*Peganum harmala*) was detected, rangeland should be enclosed	4	4	10	16	16	0.756	8
	Failure to comply with the start and stop time of grazing is one of the causes of rangeland degradation	1	5	13	17	14	0.752	9
	The principal utilization based on the grazing capacity is one of the rangeland conservation ways	2	9	11	7	21	0.748	10
	Night grazing minimize overgrazing of some plants	0	5	18	13	14	0.744	11
	Herd mobility and movements are one of the cause of rangeland degradation	0	10	13	9	18	0.740	12
	Recruitment of rangers leads to rangeland conservation	1	8	12	21	8	0.720	13
	Seizing rangelands by government bodies and organizations is one of the causes of rangeland degradation	2	5	18	13	12	0.712	14
	Avoiding early grazing is one of the rangeland conservation ways	3	8	10	17	12	0.708	15
	Providing fodder for winter feeding or droughts is one of the rangeland conservation ways	4	11	11	9	15	0.680	16
	Forming networks for pastoralists is one of the ways to support them, especially during droughts	5	6	14	16	9	0.672	17
	Supporting licensed pastoralists and legal action against unauthorized ones by government are of the rangeland conservation ways	6	11	19	2	12	0.612	18
Restoration	Implementation of grazing systems (rotation or rest rotation grazing systems) is one of the rangeland restoration ways	1	4	12	21	12	0.840	1
	The appropriate distribution of watering points is one of the rangeland restoration factors	3	5	4	12	26	0.812	2
	Timely stop of grazing will improve rangeland status	1	0	16	25	8	0.800	3
	Night grazing get livestock to graze more uniformly and get full quickly which improves rangeland condition	2	2	12	15	19	0.792	4
	Drinking frequency depends on the animal type and season	1	3	10	20	16	0.788	5
	The construction of watering points is one of the ways to restore rangelands	2	3	10	23	12	0.760	6
	The presence of a suitable number of goats in each herd, usually between 5 and 10% of the herd, improves the rangeland condition through natural seeding or grazing of some special species	1	1	13	28	7	0.756	7
	Grazing increase soil fertility because of manure	3	1	16	15	15	0.752	8
	Restoration of the fountain is one of the ways to restore rangelands	2	4	10	23	11	0.748	9
	A large number of goats in the herd may lead to rangeland degradation	5	3	4	31	7	0.728	10
	Rangelands can be divided into several camps based on the region topography (slope, aspect, elevation, hillsides direction relative to the sun)	6	3	10	18	13	0.720	11
	Light (balanced) grazing makes rangeland recovery possible	4	2	11	27	6	0.716	12
	Rangelands can be divided into several camps based on knowledge and soil types (in grazing systems)	4	7	10	18	11	0.700	13
	Rangeland enclosure increases the vegetation cover	9	3	10	15	13	0.680	14
	Some ways to restore rangelands are practices such as seeding and planting seedlings	4	8	18	15	5	0.636	15
	Harvesting rain water using natural features is one of the ways to provide drinking water for livestock	5	9	26	5	5	0.584	16
	Making pond next to the fountain is useful for livestock and rangeland	15	16	10	6	3	0.464	17

In contrast, “grazing begins based on the type and gender of livestock and the type of vegetation in different areas” and “the start of grazing depends on the health status of livestock” with the values 0.436 and 0.444, “supporting licensed pastoralists and legal action against unauthorized ones by government are of the rangeland conservation ways” and “forming networks for pastoralists is one of the ways to support them, especially during droughts” with the values 0.612 and 0.672, and “making pond next to the fountain is useful for livestock and rangeland” and “harvesting rain water using natural features is one of the ways to provide drinking water for livestock” with the values 0.464 and 0.584 were the less important items for exploitation, conservation and restoration indicators respectively.

**Figure 3:**
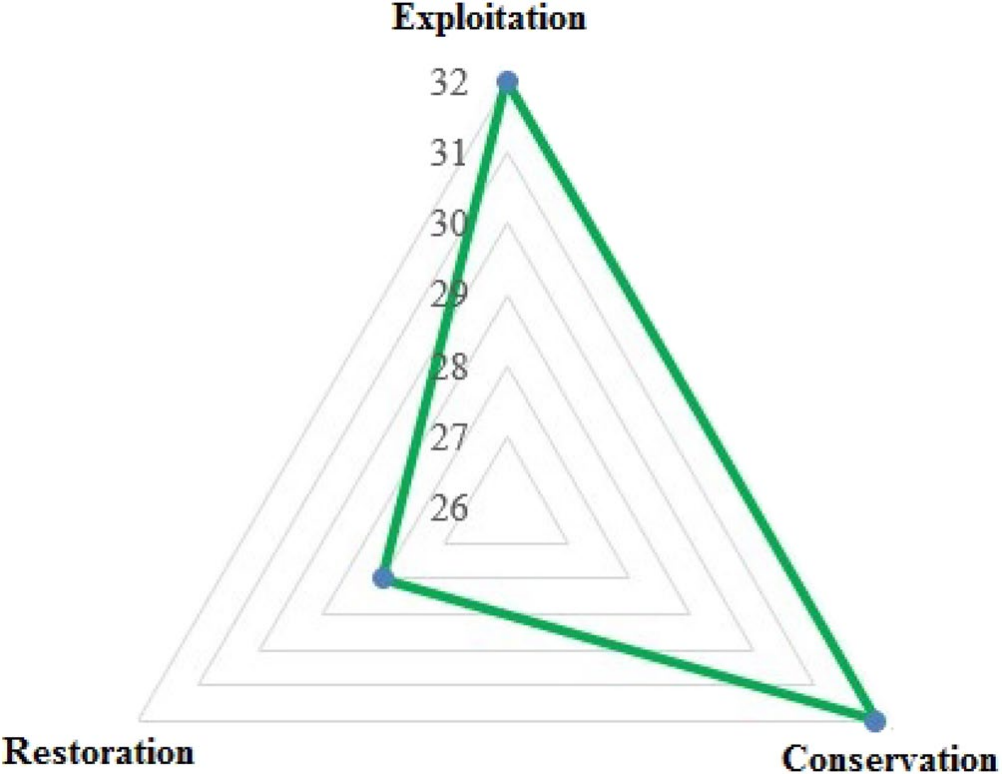
Comparison of the knowledge-based behavioral indicators of pastoralists for rangeland management.

There were significant differences between the knowledge-based behavioral indicators of pastoralists (Table 3). The results of mean rank showed that the pastoralists’ knowledge-based behavior in order from highest to lowest was related to exploitation, conservation, and restoration indicators with the values of 2.35, 2.07 and 1.58 respectively (Fig. 3).

**Table 3:** The differences between the knowledge-based behavioral indicators of pastoralists for rangeland management based on indigenous knowledge approach.

Indicator	Chi-square value	Degree of freedom	Mean rank	Rank	*p*
Exploitation	15.343	2	2.35	1	0.01
Conservation			2.07	2	
Restoration			1.58	3

The correlations between three knowledge-based behavioral indicators of pastoralists for rangeland management were significant (*p* < 0.01, Table 4). Exploitation knowledge and restoration knowledge had the strongest and weakest significant correlations respectively. There were also significant correlations between knowledge-based behavioral indicators of pastoralists and their personal qualities (*p* < 0.01, Table 4). The knowledge-based behavior of respondents for rangeland management had a positive and significant relationship with their age (r = 0.258) and a negative and significant relationship with their literacy (r = − 0.263). Experience years in pastoralism had also a positive and significant relationship with the knowledge-based behavioral indicators of pastoralists. The amount of income had also a positive and significant relationship with the knowledge-based behavioral indicators of pastoralists (r = 0.290).

**Table 4:** Correlation between the knowledge-based behavioral indicators of pastoralists and personal qualities of the respondents.

Personal qualities	The knowledge-based behavioral indicators of pastoralists							
	Exploitation		Conservation		Restoration		Total	
	r	Sig	r	Sig	r	Sig	r	Sig
Age	− 0.168	0.112	− 0.048	0.370	− 0.221	0.061	0.258*	0.035
Family size	0.269	0.316	− 0.016	0.456	0.117	0.208	0.112	0.220
Literacy	− 0.059	0.342	− 0.090	0.267	− 0.127	0.208	− 0.263*	0.028
Livestock number	0.150	0.149	− 0.134	0.176	− 0.067	0.321	0.036	0.401
Experience years in pastoralism	0.385**	0.000	0.313**	0.000	0.493**	0.000	0.370**	0.000
Income	0.230	0.054	− 0.041	0.388	0.260*	0.034	0.290*	0.021
Exploitation	–	–	0.390**	0.003	0.638**	0.000	0.896**	0.000
Conservation	0.390**	0.003	–	−	0.406**	0.000	0.600	0.000
Restoration	0.638	0.000	0.406**	0.000	–	–	0.826**	0.000

Significant correlations are shown by: *p = 0.05; **p = 0.01.

## Discussion

Today, there is an urgent need to integrate natural and social sciences for the successful management of natural resources. The emphasis is on management approaches that involve local communities in the environmental decision-making process to achieve sustainable environmental management. Local pastoralists are one of the basic components in rangeland management, playing a very important role in the interaction with other factors in the formation of the rangeland management mechanism. Pastoralists have managed their rangelands throughout history and have gained cultural potentials and valuable experiences in their interaction with nature, which should be used for the sustainable management of rangelands. In this study, we tried to reveal the knowledge-based behavior of pastoralists for rangeland management. The results of this research showed that the specialized rangeland management items used by pastoralists can be classified based on their semantic range in three indicators i.e. exploitation, conservation, and restoration.

### Pastoralists’ knowledge for rangeland exploitation

The adequate growth of palatable plants was the most important item of rangeland exploitation, which is a sign of the rangeland' readiness for grazing start based on the pastoralists’ viewpoint. In other words, the lack of annual growth of palatable plants indicates the downward trend of rangeland condition. This proves that livestock and rangeland forage quality are the first priorities for pastoralists, due to their poor living conditions and the one-dimensionality of their income sources. This findings are in line with the results of^22–24^. In fact, the decrease of dominant and palatable plants and the increase of unwanted invasive and poisonous plants are the signs of rangeland degradation, which cause emaciation and weight loss of grazing animals^15,25,26^, increase of the grazing duration and increase of the travel distance of livestock^27^. The appearance and growth of palatable plants, in addition to vegetation diversity, will increase the value of the plant composition, i.e. the higher the quality of forage in the rangelands, the higher the quality of dairy and meat products^28,29^. Rangeland readiness for grazing depends not only on vegetation growth but also on the soil. Therefore, pastoralists examine changes in the color and shape of the soil to determine the proper time at which grazing may begin^30,31^. If the soil is not ready, early grazing will lead to accelerated soil erosion in rangelands. On the other hand, pastoralists considered rangeland depletion and dust emission due to livestock trampling as the signs of the stop of grazing. Therefore, timely stop of grazing is an art that pastoralists do to conserve their rangelands. Therefore, grazing can be continued as long as, firstly, the regrowth potential of forage species especially palatable ones is maintained during grazing, secondly, livestock movement does not cause dust erosion and change in the soil shape.

Due to unaffordability of livestock management and purchasing supplementary feeding, the pastoralists are forced to start grazing early. Poverty of pastoralists and lack of non-husbandry sources of income have caused their livelihood and well-being to depend heavily on livestock productivity and rangeland forage. This has intensified due to severe climatic fluctuations and droughts in the last decade. Pastoralists are forced to move their herds early from winter rangelands to summer rangelands due to the lack of forage production and challenges of providing drinking water for livestock. Meanwhile, the extreme fluctuations in the supply and price of fodder in the market have made rangeland-based pastoralism uneconomical. Nevertheless, some pastoralists are still engaged in rangeland-based pastoralism according to their old traditions.

### Pastoralists’ knowledge for rangeland conservation

The results obtained from the conservation knowledge of pastoralists showed that they have considered holding meetings by elders and reducing the number of pastoralists during droughts as the most important ways to conserve rangelands. Pastoralists in the studied area believed that rangeland conservation starts with themselves as the main beneficiaries of the rangeland. The participation and solidarity of pastoralists makes it possible to be effective in the rangeland conservation while preventing rangeland degradation factors. On the other hand, it is possible to prevent the unauthorized pastoralists from entering the pastoral units in order to avoid overgrazing (the excessive livestock numbers over the grazing capacity), which is one of the most important factors of rangeland degradation.

Due to the recent droughts and severe temperature fluctuations, pastoralists have become more vulnerable in the studied area. The high climatic variability has caused great fluctuations in the supply of rangeland forage and subsequently livestock production, becoming a challenge for livestock management^2,32^. One of the ways to conserve rangelands is to reduce the number of exploiters, according to the pastoralists themselves. Small-scale pastoralists who have less than 100 heads of livestock should hand over their livestock to other pastoralists according to the agreement of the parties. This can have various consequences, such as reducing the animal movement in rangeland, reducing the loss of forage, reducing the number of the pastoral units, and increasing the grazing area of the herd and the proper distribution of livestock in the rangeland. By reducing the number of pastoralists, it is possible to focus more on the drinking water management of livestock especially during droughts. Reducing the number of livestock during droughts through the establishment of a fattening system and selling livestock in local and regional markets or the guaranteed purchase of surplus livestock by the government can reduce the grazing pressure on rangelands. Some pastoralists considered another option, the change of herd composition and matching it with the type of leftover forage in the rangeland. This finding is in line with the results of other researchers^33,34^. Removal of old animals and surplus males by selling them may change the herd composition. This finding is consistent with other researches^35^.

### Pastoralists’ knowledge for rangeland restoration

Based on the results, pastoralists implement restoration strategies for rangeland recovery which is in the line with the results of other researchers (36). The most important restoration strategies used by pastoralists was rangeland management i.e. rotational grazing systems and resting some parts of the rangeland (enclosure), which are consistent with the results of other researchers^37,38^. Enclosing a portion of rangeland is the most common approach for vegetation restoration and recovery. The pastoralists believed that the implementation of the rest rotational grazing system and exclusion of livestock from a portion of rangeland help to restore the vegetation and soil and create an opportunity for the natural recovery of vegetation. Water resources management was another restoration strategy used by the pastoralists of the region. The proper distribution of watering points and supply of qualitative drinking water for livestock are the basis for the better movement of herd and correct distribution of livestock, which lead to uniform utilization of forage in rangelands. These results are consistent with the other researches^27,33,39–42^. In other words, pastoralists have a lot of knowledge on grazing management and herding, which can be used to restore degraded rangelands^27,37,43,44^. The high temperature is a limiting factor for grazing during the day. Therefore, pastoralists use night grazing to solve this problem. In the night grazing system, due to the cooler temperatures compared to the day, the livestock can travel a greater distance without drinking water and it is possible to have better use of the rangeland forage.

### The relationships between pastoralists’ personal qualities and their knowledge for rangeland management

As the results showed, the behavior of pastoralists towards rangeland management changed and their knowledge level increased as their age and experience years in pastoralism increased. Aging is along with the increase in experience, observation and different trials and errors, which results in the higher level of indigenous and experimental knowledge in older pastoralists than younger ones^45,46^. In addition, education, income and trust in the government's participation in the rangeland restoration and conservation have a positive effect on the conservation knowledge of pastoralists^47^. The interactions and continuous communication, while expanding their social network, provide the basis for increasing the knowledge of pastoralists. On the other hand, the poor living condition of elders has caused young people to have less desire for pastoralist life. Despite the low literacy level in the elderly, they have more information regarding the dimensions of rangeland management in the form of indigenous knowledge. Therefore, they may not be able to write down their knowledge, but they have the ability and skill to transfer their knowledge to others. Also, the findings indicated that the higher knowledge level of pastoralists in rangeland management, the higher income from animal husbandry. The results of other studies have confirmed the obtained findings^36,47^. For example, Maasai pastoralists in Kenya use traditional methods to manage livestock nutrition, diseases, breeding and protection against predators and other accidental events^10^. Forage storage during the rainy season and its use during the droughts and seasonal movement of herds are two important traditional grazing strategies used by pastoralists^41^. Common free grazing of rangelands, optimal use of rangeland, division of the herd based on plant species in rangeland, and seasonal assessment of rangeland condition are of the traditional principles of management among pastoralists^15^. Other strategies for grazing management include purchasing supplementary feed during droughts and reducing the number of livestock in sensitive times^42^. Health care and purchasing supplementary feed during droughts and choosing suitable rams with the characteristics desired by the pastoralists have been of the rangeland management practices among the Botswana pastoralists^48^. With the increase in the number of livestock, the pastoralists tries to meet their needs based on individual knowledge, forcing them to seek to acquire and increase their knowledge and information in order to reduce the costs of animal husbandry and animal maintenance, especially for health care, veterinary medicine and animal pests.

Although in this research, the analysis of a number of items based on separate axes was examined and evaluated to prove the compatible traditional techniques and methods, but the results show that these traditional techniques and methods are compatible with sustainable development. It seems that this classification of items is able to pay attention and emphasize components such as grazing methods, grazing systems, determination of rangeland capacity, production estimation, allowable use and pastoral units’ characteristics and provide a suitable model for sustainable conservation and exploitation of rangelands. The summary of the opinions of the pastoralists shows that social structure and geographical conditions play an effective role in the formation and development of items. Although many believe that with the introduction of modernity into the social system, we are witnessing the breaking of emotional bonds and the neglect of identity roots in different social layers, it is recommended that for the implementation of cooperative projects and activities to achieve a sustainable management in rangelands, the integration of local ecological knowledge with official knowledge along with the promotion of the social status of the pastoralists should be considered by the experts.

## Conclusion

Indigenous knowledge is an effective knowledge in rangeland management, including exploitation, conservation, and restoration. The objective manifestation of this claim is the better condition of region rangelands with indigenous knowledge management compared to rangelands managed with modern knowledge. Undoubtedly, the prevailing problems in the pastoralism rangelands have a complex and sometimes completely unknown nature, and prescribing a similar prescription will not answer the degradation process prevailing in the rangelands. Consequently, indigenous knowledge approach can be considered as an effective component in the rangeland management, and perhaps it can be said that the compatible integration between local knowledge and modern knowledge can lead to the strengthening of the positive effects of both management systems and results to an evolution in the condition of pastoralism rangelands.

## Data Availability

The datasets used and/or analyzed during the current study are available from the corresponding author on reasonable request.
